# Biomimetic Hierarchically Arranged Nanofibrous Structures Resembling the Architecture and the Passive Mechanical Properties of Skeletal Muscles: A Step Forward Toward Artificial Muscle

**DOI:** 10.3389/fbioe.2020.00767

**Published:** 2020-07-16

**Authors:** Carlo Gotti, Alberto Sensini, Gianmaria Fornaia, Chiara Gualandi, Andrea Zucchelli, Maria Letizia Focarete

**Affiliations:** ^1^Department of Industrial Engineering, Alma Mater Studiorum-Università di Bologna, Bologna, Italy; ^2^Advanced Mechanics and Materials–Interdepartmental Center for Industrial Research (CIRI-MAM), Alma Mater Studiorum-Università di Bologna, Bologna, Italy; ^3^Department of Chemistry “G. Ciamician” and National Interuniversity Consortium of Materials Science and Technology, Bologna Research Unit, Alma Mater Studiorum-Università di Bologna, Bologna, Italy; ^4^Health Sciences and Technologies–Interdepartmental Center for Industrial Research (CIRI-HST), Alma Mater Studiorum-Università di Bologna, Bologna, Italy

**Keywords:** electrospinning, hierarchical structures, artificial muscle, bioinspired structures, polyurethane, nanofibers, biomechanical modeling

## Abstract

Skeletal muscles are considered to date the best existing actuator in nature thanks to their hierarchical multiscale fibrous structure capable to enhance their strength and contractile performances. In recent years, driven by the growing of the soft robotics and tissue-engineering research field, many biomimetic soft actuators and scaffolds were designed by taking inspiration from the biological skeletal muscle. In this work we used the electrospinning technique to develop a hierarchically arranged nanofibrous structure resembling the morphology and passive biomechanical properties of skeletal muscles. To mimic the passive properties of muscle, a low-modulus polyurethane was used. Several electrospun structures (mats, bundles, and a muscle-like assembly) were produced with different internal 3D arrangements of the nanofibers. A thermal characterization through thermogravimetric and differential scanning calorimetry analysis investigated the physico-chemical properties of the material. The multiscale morphological similarities with the biological counterpart were verified by means of scanning electron microscopy investigation. The tensile tests on the different electrospun samples revealed that the muscle-like assembly presented slightly higher strength and stiffness compared to the skeletal muscle ones. Moreover, mathematical models of the mechanical behavior of the nanofibrous structures were successfully developed, allowing to better investigate the relationships between structure and mechanics of the samples. The promising results suggest the suitability of this hierarchical electrospun nanofibrous structure for applications in regenerative medicine and, if combined with active materials, in soft actuators for robotic.

## Introduction

Skeletal muscle is one of the most fascinating tissues designed by nature. It is the major actuator of the animal body, due to its contractile properties. This kind of muscles is referred as “skeletal” because it is linked to the skeleton at least on one side. Its main functions are sustaining loads, providing mobility, supporting the body and generating heat (Shahinpoor et al., 2007). In order to achieve all these functions, evolution led to the organization of the skeletal muscular tissue in a complex fibrous hierarchical structure (Fung, 1993; Frontera and Ochala, 2015). The building block of this morphology is the muscle fiber (myofiber), a polynucleated fiber-like cell embedding several parallel smaller fibrillar units, called myofibrils. Myofibrils consist in repeated sections named sarcomeres, composed of fibrous proteins chains (i.e., actin and myosin) able to slide over each other, generating the muscle contraction (Rassier, 2010). Muscle fibers then aggregates in bundles of increasing complexity, covered by randomly arranged collagenous membranes (i.e., endomysium, epimysium, and perimysium) (Light and Champion, 1984; Gillies and Lieber, 2011). The mechanical properties of skeletal muscles can be divided in active and passive ones. The former are linked to the shortening of the sarcomere caused by the sliding of the actin/myosin filaments during the muscular contraction; the latter instead, depend only on the tissue mechanical response while an external force stretches the inactivated muscle (Herbert, 1988). The effect of this passive stretching on the nanofibrous arrangement of the muscles causes their typical non-linear elastic behavior (Herbert, 1988; Full and Meijer, 2000; Toursel et al., 2002; Takaza et al., 2012). The possibility to replicate the physiological and morphological muscular structure of the native muscle with an artificial one opens great opportunities in the fields of tissue regeneration and soft robotics. To develop a bioinspired artificial muscle two main components are needed: an active one, suitable to replicate the function of actin and myosin, and a proper passive bulk material capable to support and follow the mechanical contraction/extension promoted by the active one. In particular, the passive component should be able to reproduce the mechanical properties of muscles. However, due to the high variability of muscle tissue and the lack of standards for testing, native muscle mechanical properties reported in the literature vary in a wide range. Calculated through tensile tests on inactivated muscles, the failure stress was reported in a range of 70–800 kPa with a failure strain of 30–60% and an elastic modulus of 30–8000 kPa (Moss and Halpern, 1977; Kovanen et al., 1984; Lakie and Robson, 1988; Lieber et al., 1991; Wolff et al., 2006; Hoang et al., 2007; Shanshan et al., 2010; Schleifenbaum et al., 2016).

To mimic such soft and stretchable properties, polyurethanes (PUs) are promising materials for building artificial muscles. This class of polymers has the peculiarity of displaying a wide range of mechanical performance. By properly engineering their chemical composition in terms of hard-soft segments, it is possible to synthetize elastomeric materials that have been widely employed both for muscle tissue engineering and for soft robotic (Asaka and Okuzaki, 2014). Moreover, they are also biocompatible and, depending on the chemical structure, biodegradable (Chen et al., 2010). Among the different manufacturing techniques used to produce PU-based scaffolds and actuators (Janik and Marzec, 2015; Smith and Grande, 2015), the electrospinning technology has been demonstrated to be a powerful technique to build 3D nanofibrous soft tissues-inspired structures. This is achieved by engineering the experimental setups producing mats of nanofibers that can be assembled in bundles/yarns or higher hierarchical levels (Jiang et al., 2015; Sensini et al., 2017, 2018c, 2019b). Focusing on electrospun PU nanofibers, an high deformability has been reported (failure strain = 160–400%; Pedicini and Farris, 2003; Yao et al., 2008; Erdem et al., 2015; Chernonosova et al., 2018)along with high elasticity (elastic modulus = 1.5–4 MPa; Yao et al., 2008). Few works have dealt with proposing electrospun PU scaffolds for muscle reconstruction, often limiting themselves to two-dimensional patches to convey *in vitro* proliferation of myoblasts (Riboldi et al., 2005; Liao et al., 2008; Caracciolo et al., 2010).

In the soft actuators production, electrospun PUs were processed in different levels of hierarchical aggregation. Starting from simplified structures, aimed at obtaining a bending actuator, Kwon et al. (2013) proposed mats of PU nanofibers covered with a vapor-deposited sheath of electroactive poly(3,4-ethylenedioxy thiophene) (PEDOT). Analogous actuators were realized by Ebadi et al. (2019a, b), by coating mats of electrospun PU nanofibers, doped with p−toluenesulfonate (pTS) or bis(trifluoromethylsulfonyl)imide (TFSI), with polypirrole (PPy). Increasing the structures complexity, Kang Gu et al. (2009) proposed an artificial myofiber made of parallelly arranged PU nanofibers grouped together in a bundle, coated with polyaniline (PANi). More recently, Meng et al. (2019) developed a yarn actuator by electrospinning a blend of aligned PU nanofibers loaded with carbon nanotubes (CNTs).

The above described solutions have potentiality to work as scaffolds, thanks to the cellular tendency to grow up along aligned stretchable PU nanofibers, and as actuators, thanks to the combination of the passive elastomeric matrix of PU with an electro-active component. However, the possibility to achieve the challenging objective of faithfully reproducing the 3D hierarchical morphology of the native muscle is expected to bring further improvements in the overall properties and functionalities of these kind of constructs that will have important outcomes both in tissue engineering and soft robotics.

This work reports a method for developing an innovative PU-based hierarchical nanofibrous electrospun structure (HNES) aimed at reproducing the morphology and the passive mechanical properties of skeletal muscles. This muscle-like assembly was produced by grouping several bundles of aligned fibers tighten together by an external membrane of electrospun random fibers. Parallelly, bundles and electrospun 2D mats substructures were also produced and characterized for comparison. Morphological and mechanical characterization of the different structures was performed and, to complete the multiscale investigation of such nanofibrous structures, numerical models of their mechanical behavior were developed. The proposed hierarchical muscle-inspired structure paves the way for future research in the field of artificial muscle tissue engineering and soft robotics.

## Materials and Methods

### Materials

Poly[4,4′-methylenebis(phenyl isocyanate)-alt-1,4-butanediol/di(propylene glycol)/ polycaprolactone], a PU constituted by hard segments of 4,4′-methylenebis(phenyl isocyanate) (MDI) and polyether-ester soft segments, was purchased from Merck (M_w_ = 56,000 g/mol, PDI = 1,8, by Gel Permeation Chromatography in THF calibrated with polystyrene standard). Tetrahydrofuran (THF, 99.9%) and N,N-Dimethylformamide (DMF, 99.8%) were supplied by Merk and used without further purification.

### Electrospun Structures Preparation

The solution for electrospinning was prepared by dissolving PU at a concentration of 25% w/v in a mixed solvent of THF:DMF = 70:30 (v/v). A commercial electrospinning machine (Spinbow srl, Bologna, Italy) equipped with a linear sliding spinneret with four needles and a rotating drum collector was used. The needles were fed, through PTFE tubes, by four syringes carrying the polymeric solution with a flow rate controlled by a syringe pump (KD Scientific 200 series, IL, United States). The sliding spinneret had a linear excursion of 180 mm along the collector, with a speed of 1500 mm min^–1^. PU solution was electrospun by applying the following processing conditions: applied voltage = 23 kV, feed rate = 0.3 ml h^–1^, needles-collector distance = 180 mm, needles inner diameter = 0.51 mm. The process was performed at room temperature and relative humidity 30–40%.

**FIGURE 1:**
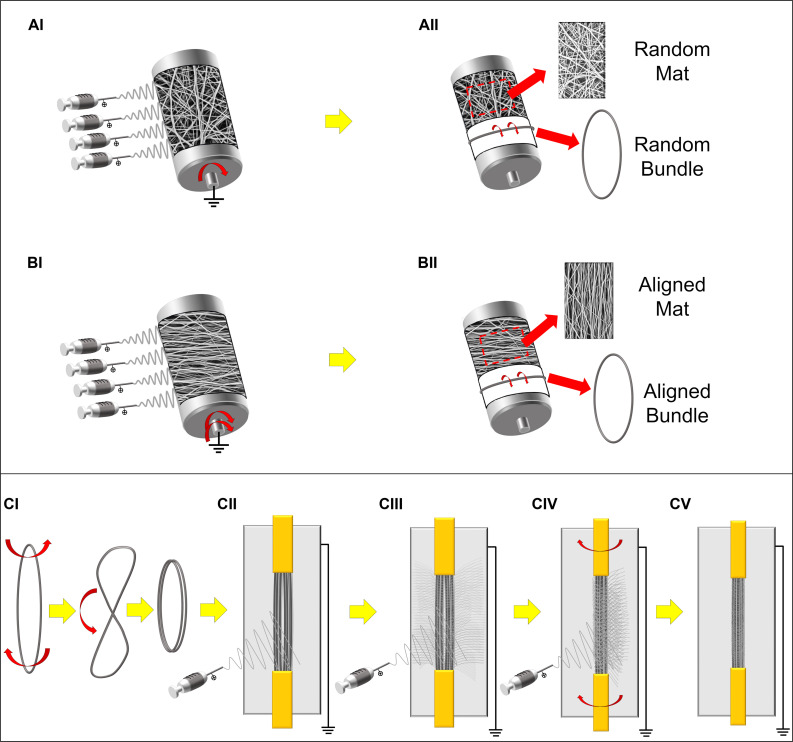
Schematic representation of the electrospun setups and samples preparation. **(A)** Production of the PU random mats and bundles: **(AI)** random nanofibers were electrospun on the low-speed rotating drum collector. **(AII)** The mats were then either removed and cut in rectangular specimens or, cut in stripes, wrapped up and finally pulled out from the drum producing ring-shaped bundles of random nanofibers. **(B)** Production of the PU aligned mats and bundles: **(BI)** aligned nanofibers were electrospun on the high-speed rotating drum collector. **(BII)** The mats were then either removed and cut in rectangular specimens or cut in stripes, wrapped up and finally pulled out from the drum producing ring-shaped bundles of axially aligned nanofibers. **(C)** Preparation of the HNES: **(CI)** Two aligned bundles were twisted in the middle and bended and **(CII)** fixed in a custom-made electrospinning rotating setup with a flat collector in the rear part; **(CIII,CIV)** alternating periods of stasis and rotation during the PU electrospinning, **(CV)** the external epimysium-like membrane was produced on the final HNES.

Electrospun PU nanofibers that reproduce the skeletal muscle myofibrils (mean diameter = 1 micrometer; Shahinpoor et al., 2007), were collected in form of non-woven flat mat on the aluminum drum collector (length = 405 mm, diameter = 150 mm). Fiber directionality was controlled by the drum peripheral speed. In detail, a random nanofibers distribution was obtained by setting 50 rpm, corresponding to a peripheral speed of 0.4 m s^–1^ (Figure 1AI); an aligned nanofiber distribution was obtained at 2500 rpm (peripheral speed = 19.6 m s^–1^) (Figure 1BI). To easily detach the mats, the drum was covered with a sheet of polyethylene (PE) coated paper (Turconi S.p.A, Ceriano Laghetto, Italy). After 3 h of electrospinning the mat thickness was in the range 10–20 μm.

To reproduce a muscle fiber, bundles of nanofibers were prepared by following a previously reported procedure (Sensini et al., 2019a). Briefly, once obtained the random and aligned flat mats as described above, several stripes of the electrospun membranes were cut circumferentially directly on the drum collector. The stripes were then manually rolled up and pulled out from the drum, obtaining ring shaped bundles (cross-sectional diameter = 350–500 μm) (Figures 1AII, BII).

Bundles of aligned fibers were adopted to realize a muscle-like assembly. HNES were produced using a previously reported procedure (Sensini et al., 2020). Briefly, two parallelly arranged bundles of aligned nanofibers were twisted once in the middle and bended on themselves (Figure 1CI). The bundles were subsequently grouped together using a nanofibrous membrane, obtained with a recently developed electrospinning procedure (Sensini et al., 2020). This membrane was applied to pack and tight together the bundles as the biologic membrane epimysium does in the skeletal muscle (Frontera and Ochala, 2015; Sensini et al., 2018b).

The same electrospinning parameters described above were applied to electrospin the membrane by using a custom-made electrospinning machine. The electrospinning apparatus was composed of a high-voltage power supply (FuG Elektronik GmbH, Schechen, Germany), one syringe pump (KD Scientific Legato 100, Illinois, United States), and a glass syringe containing the PU solution, connected to a stainless-steel blunt-ended needle (inner diameter = 0.51 mm) by a PTFE tube. A flat aluminum collector plate (200 mm high and 50 mm wide) was placed behind the bundles. The group of bundles were intermittently put in rotation (∼20 rpm for 1 min every 10 min), during a 90 minutes long electrospinning process (Figures1CII–CV). At the end of the process the HNES showed two looped extremities.

### Thermal Characterization

Thermogravimetric analyses (TGA) were carried out using a TGA Q500 thermogravimetric analyzer (TA Instruments, United States). Analyses were performed from RT to 700°C, at a heating rate of 10°C min^–1^, under nitrogen flow.

Differential Scanning Calorimetry (DSC) was carried out with a DSC Q100 (TA Instruments), equipped with a refrigerated cooling system (RCS). Samples were subjected to two heating scans at 20°C/min and one controlled cooling at 10°C/min, applied between the heating scans. The glass transition temperature (T_g_) was taken at half-height of the glass transition heat capacity step whereas the melting temperature (T_m_) was taken at the peak maximum of melting endotherm.

### Morphological Characterization

The specimen surface was observed with a Scanning Electron Microscope (SEM, Phenom Pro-X, PhenomWorld, Eindhoven, Netherlands), applying 10 kV on gold sputter-coated samples. ImageJ (Liu, 1991) was used to measure about 200 nanofiber diameters and data are provided as mean and standard deviation. The bundles and HNES diameters were taken with an optical microscope (Axioskop, Zeiss, Pleasanton, CA, United States) equipped with a camera (AxioCam MRc, Zeiss, Pleasanton, CA, United States), and data are provided as mean and standard deviation of 20 measures. Fiber orientation was evaluated by using the Directionality plugin of ImageJ (Schindelin et al., 2012; Schneider et al., 2012). This approach allowed to quantify the amount of nanofibers within a given angle from the axis using a Local Gradient Orientation method, following a previously validated procedure (Sensini et al., 2018a, 2019a). For each specimen the analysis was performed on five images (magnification = 3000x).

### Mechanical Characterization

**FIGURE 2:**
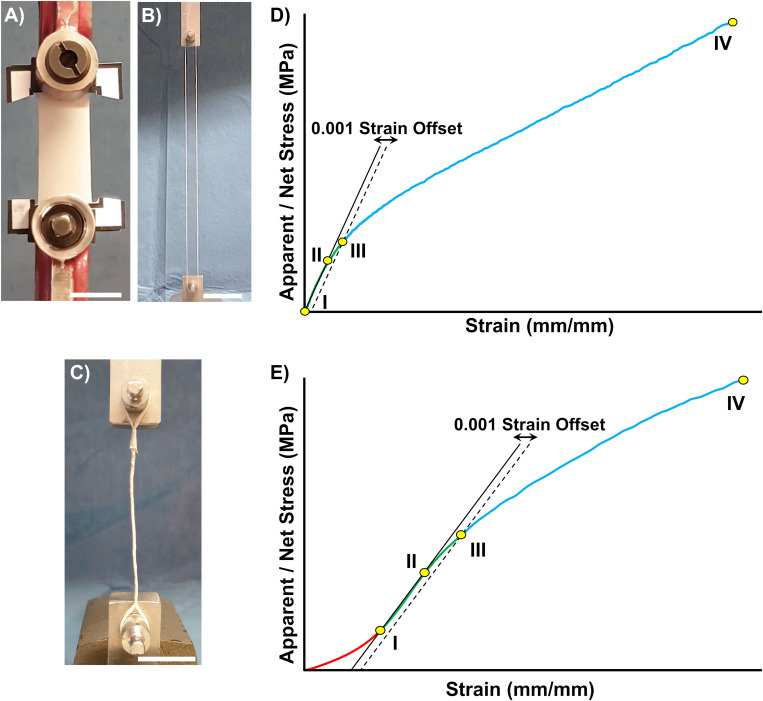
Mechanical characterization setup and data post processing. Tensile test configuration for: **(A)** Mats, **(B)** Bundles, and **(C)** HNES (scale bars = 20 mm). The stress-strain curves showed different behaviors for the random **(D)**, aligned and HNES samples **(E)**, requiring two slightly different approaches to treat the mechanical data. **(D)** Typical stress-strain curve of random nanofiber specimens. A first linear regression (solid line) was applied from 0 N **(DI)** to a starting yield stress (**DII** arbitrary chosen). A second line parallel to the first regression was drawn with an offset of 0.001 strain (dashed line) **(DIII)**. The limit of proportionality (σ_Y_) was defined with the 0.001 strain offset criterion as the intersection between the latter line and the stress-strain curve **(DIII)**. The elastic modulus **(E)** was calculated as the slope of a new regression line between **(DI,DIII)**. The failure stress (σ_F_) **(DIV)** was identified as the highest stress in the entire curve. The unit work to yield (L_Y_) and to failure (L_F_) were calculated as the integrals under the curves (with the method of trapezoids). The asymptotic stiffness (AS) was calculated as slope of a regression between **(DIII,DIV)**. **(E)** Typical stress-strain curve of the aligned nanofiber specimens and HNES. The starting point of the linear region **(EI)** was identified as the point corresponding to the 20% of failure stress for mats and bundles (25% for HNES). The initial toe region (from 0 N to **EI**) was disregarded. The curves were then elaborated as previously described in **(D)**.

The mechanical tensile characterization of the electrospun specimens was carried out with a material testing machine (Mod. 4465, Instron, Norwood, United States) with a ± 100 N load cell (Instron, Norwood, United States). The test machine worked under displacement control to obtain an average strain rate of 0.33% s^–1^. For each type of specimen (i.e., mats, bundles and HNES) the grip configuration was optimized in order to guarantee an accurate estimation of the specimen mechanical performances and to reduce the stress concentrations (Figures 2A–C).

In the case of random and aligned nanofibrous mats ten replicates each were tested and, to guarantee a proper centering of the specimen in the machine, each mat was fixed in a rectangular paper frame (outer dimension = 67 × 47 mm; inner dimension = 45 × 25 mm) with cyanoacrylate glue, using a previously developed procedure (Maccaferri et al., 2019). The specimen was then fixed at the extremities in the machine clamps (Instron, Norwood, United States). Before starting the test, the sides of the paper frame were cut (Figure 2A). The effective specimen dimensions for the random mats were 45 × 20 mm (i.e., length × width). The aligned mats instead, showed a slightly shrink of 5 mm after frame cutting, resulting in a final gauge length of 40 × 20 mm. The thickness of each specimen was measured as mean and standard deviation between 30 measures, using a digital indicator (ALPA, Pontoglio, Italy) with a 0.65 N preload, a resolution of 1 micrometer, a maximum error of 4 micrometers and a repeatability of 2 μm.

The mechanical performances of the ring bundles were tested using a monotonic ramp to break (consistently to the ASTM D1414 Standard) on ten replicates for each type of sample. The random bundles gauge length was 228 mm, while the aligned bundles gauge length was 197.5 mm, caused by the specimen shrinkage after detachment from the drum collector. Finally, three HNES, resulting in a final gauge length of 98.0 mm, were tested with the same bundles procedure and setup (Figure 2C).

The force-displacement curves were converted to stress-strain graphs using two different approaches. In the first one, the apparent stress, calculated by dividing the force by the cross-sectional area of the specimen measured before the test, was plotted against strain, whereas in the second one, the net stress was used, in order to determine the mechanical properties of the specimen independently from its porosity. The net stress was calculated by dividing the apparent stress by the volume fraction (*v*) of the specimens. The volume fraction (*v*) was calculated by using the equation:

(1) $$v=\frac{w}{\left(L\cdot A\cdot \rho \right)}$$

Where w is the weight of the specimen, L is length of the specimen, A is the cross-sectional area of the specimen, ρ is the density of the raw material (PU = 1.18 g/cm^3^).

The following indicators were considered (Figures 2D,E): Yield Stress (σ_Y_), Yield Strain (ε_Y_), Elastic Modulus (E), Asymptotic Stiffness (AS), Failure Force (F_F_), Failure Stress (σ_F_), Failure Strain (ε_F_), Unit Work to Yield (L_Y_), Unit Work to Failure (L_F_).

### Statistical Analysis

The significance of differences between the apparent mechanical properties for the mats (n = 10) and bundles (*n* = 10) with the same nanofibers orientation (i.e., random or aligned), and for the aligned bundles and the HNES (*n* = 3) was assessed with an unpaired parametric *t*-test with Welch’s correction. With the same procedure, the net mechanical properties were compared. Instead, the comparison between the apparent and net mechanical properties of the same sample (i.e., random mats, aligned mats, random bundles, aligned bundles, and HNES) was assessed with a ratio paired parametric *t*-test.

### Mechanical Modeling

In addition to the conventional analysis of stress-strain curves, some mathematical relationships were also considered in order to obtain a generalized modeling of the mechanical behavior of the investigated materials. For modeling purpose, the stress-strain data have been limited in strain before the failure of the samples (strain limit called ε^∗^). Moreover, the modeling has been applied only to the net stress-strain data, that resulted less scattered compared to the apparent ones, in line with previous results (Maccaferri et al., 2019). Two mathematical models have been considered starting from the net stress-strain curves of the specimens previously described. The first model, here called Z-Model, was introduced in Maccaferri et al. (2019) for the analysis of the mechanical behavior of polyamide nanofibrous mats and is a linear combination of a linear term and a non-linear-asymptotic term as follows:

(2) $${\text{σ}}_{Z}(\text{ε})={\text{σ}}_{\text{Z},Lin}(\text{ε})+{\text{σ}}_{\text{Z},Lin}(\text{ε})=(a_{Z}\text{ε}+b_{Z})+(-b_{Z}e^{-c_{z}\text{ε}})=a_{Z}\text{ε}+b_{Z}(1-e^{-c_{z}\text{ε}})$$

The second model, here called F-Model, was previously introduced for the study of mechanical behavior of biological soft tissues (Fung, 1967; Vladimir and Mow, 1980) and is a linear combination of a constant and an exponential term as follows:

(3) $$\sigma_{F}\,\left(\varepsilon \right)=\left(-B_{F}\right)+B_{F}\,e^{C_{F}\,\varepsilon }=B_{F}\,\left(e^{C_{F}\,\varepsilon }-1\right)$$

**FIGURE 3:**
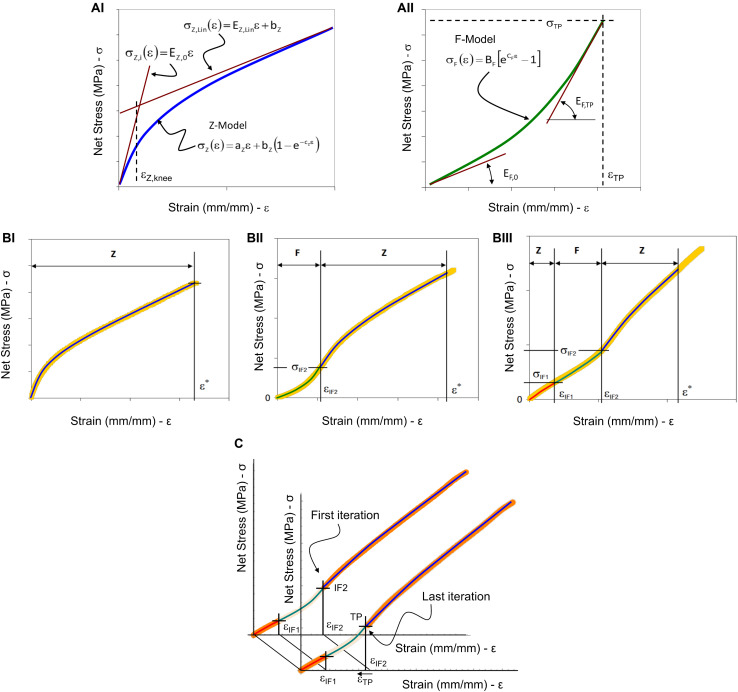
Graphical representation of the model fitting parameters and procedure. **(A)** Two models were used to analyze the tensile test results: in **(AI)** the Z-Model is represented with the initial and the linear asymptotic tangent lines, and with the strain-knee point used to evaluate the transition from the non-linear trend to the linear-asymptotic one; in **(AII)** the F-Model is represented with the two tangent lines at the initial and final strain position. **(B)** The three cases of experimental stress-strain curves obtained by tensile testing of nanofibrous materials and the representation of model combinations: **(BI)** The case where Z-Model alone is able to describe the experimental data (letter “Z”); **(BII)** The case where F-Model and Z-Model are combined at the IF2 flexural point; **(BIII)** The case where there are two flexural points and it is necessary to apply twice the Z-Model and once the F-Model. **(C)** Iterative procedure to define the transition point and the Z-Model fitting of the last part of the aligned specimens curve. The F-Model was used to fit the curve from the first inflection point (IF1) up to the second inflection point (IF2) (green line). In a preliminary step, the second Z-Model was applied to fit the curve from the IF2 up to the end of the strain-softening region (blue line). Then, using an iterative backward least-squares method optimization between the F-Model and the second Z-Model, the transition point (TP) was defined and chosen as new joining point between the F-Model and the second Z-Model.

In Figure 3A the two models are graphically represented with some additional characteristic curves and points.

For both models it is interesting to analyze the trend of the slope of the tangent to the curve by the derivative of the model respect the strain. For the Z-Model it is possible to obtain the following relation:

(4) $$\frac{d\,\sigma_{Z}}{d\,\varepsilon }\,\left(\varepsilon \right)=E_{Z}\,\left(\varepsilon \right)=a_{Z}+b_{Z}\,c_{Z}\,e^{-c_{z}\,\varepsilon }$$

which clearly shows that the slope, also referred as instantaneous elastic modulus (E_Z_), is continuously decreasing from an initial value when the strain is zero and it tends to an asymptotic value. Such a behavior can be described by considering the two main representative values for the E_Z_: the initial value, E_Z,0_ (calculatedat ε = 0), and the elastic modulus of the linear asymptotic trend, E_Z,Lin_. These two values can be calculated as follows:

(5) $$E_{Z,0}=\underset{\varepsilon \to 0}{L\,i\,m}E_{Z}\,\left(\varepsilon \right)=a_{Z}+b_{Z}\,c_{Z}$$

(6) $$E_{Z,\mathrm{Lin}}=\underset{\varepsilon \to \infty }{L\,i\,m}E_{Z}\,\left(\varepsilon \right)=a_{Z}$$

Using the expressions now found, it is possible to write the equations of the two lines describing, respectively, the tangent to Model-Z at the initial point and the linear-asymptotic trend. The two lines have the following equations and they are represented in Figure 3AI:

(7) $$\sigma_{Z,i}\,\left(\varepsilon \right)=E_{Z,0}\,\varepsilon$$

(8) $$\sigma_{Z,\mathrm{Lin}}\,\left(\varepsilon \right)=E_{Z,\mathrm{Lin}}\,\varepsilon +b$$

The intersection of the two lines allows to calculate the knee point that is defined as the transition point from the initial non-linearity to the asymptotic linearity of Z-Model. As it was previously demonstrated (Maccaferri et al., 2020), the point of intersection between the two lines gives the strain at knee of the Z-Model:

(9) $$\varepsilon_{Z,\mathrm{knee}}=\frac{1}{c_{Z}}$$

An additional parameter which is useful to study the mechanical behavior of the material is the ratio between the initial and the linear-asymptotic elastic modulus which is calculated as follows:

(10) $${△}_{Z}=\frac{E_{Z,\mathrm{Lin}}-E_{Z,0}}{E_{Z,0}}=-\frac{b_{Z}\,c_{Z}}{a_{Z}+b_{Z}\,c_{Z}}$$

For the F-Model the slope of the tangent to the model is continuously increasing from an initial value up to the value at the strain point where the model is limited, here named as Transition Point (TP). This point in the modeling of the soft biological collagenous tissues is defined as the end value of the non-linear toe region, in which the collagen fibers are relaxed and crimped, where the majority of the collagen fibers are stretched and aligned (Tanaka et al., 2011; Lee et al., 2017). In this study the TP is used to define the point in which the nanofibers have completely recovered from their shrunk status.

The instantaneous elastic modulus can be calculated as follows:

(11) $$\frac{d\,\sigma_{F}}{d\,\varepsilon }\,\left(\varepsilon \right)=E_{F}\,\left(\varepsilon \right)=B_{F}\,C_{F}\,e^{C_{F}\,\varepsilon }$$

which is an exponential function. From this relation it is possible to calculate the slope of the tangent to the curve at the initial and final strain values:

(12) $$E_{F,0}=\underset{\varepsilon \to 0}{L\,i\,m}E_{F}\,\left(\varepsilon \right)=B_{F}\,C_{F}$$

(13) $$E_{F,\mathrm{TP}}=\underset{\varepsilon \to \varepsilon_{\text{TP}}}{L\,i\,m}E_{F}\,\left(\varepsilon \right)=B_{F}\,C_{F}\,e^{C_{F}\,\varepsilon_{\text{TP}}}$$

Also, in the case of the F-Model it is useful to calculate the elastic modulus variation between the initial and the final point. In the case of F-Model, the variation of the elastic modulus depends on the endpoint ε_TP_. The relationship that defines this ratio is the following:

(14) $$\Delta_{\text{TP}}=\frac{E_{F,\mathrm{TP}}-E_{F,0}}{E_{F,0}}=\frac{B_{F}\,C_{F}\,e^{C_{F}\,\varepsilon_{\text{TP}}}-B_{F}\,C_{F}}{B_{F}\,C_{F}}=e^{C_{F}\,\varepsilon_{\text{TP}}}-1$$

The previously reported models, and their respective main characteristics, will be used to study the mechanical response of the nanofibrous materials and in particular to evaluate the impact of nanofibers arrangement in the material (i.e., random and aligned) and the geometrical structuring (i.e., mats, bundles and HNES).

These models are used to describe the three main types of curves that have been found experimentally. In Figure 3B the three types of curves are presented with some characteristic strain values, which are used to divide the curves into parts that can be successfully described by the proper combination of the two models. Figure 3BI shows that the Z-Model can adequately describe the whole curve up to the ε^∗^ limit. In the mathematical modeling of soft tissues, the value called Inflection Point (IF) is generally used to describe, a point of flex in the stress-strain curve of a material, that corresponds to an irreversible stretch of the internal fibrous structure (Tanaka et al., 2011; Lee et al., 2017). For the curve in Figure 3BII the presence of an inflection point at ε_IF2_ is observed. This point is used to divide the curve into two parts, which can be descended with F-Model and Z-Model, respectively. Finally, in the curve in Figure 3BIII it is possible to identify two strain values for which there are two inflection points: ε_IF1_ and ε_IF2_. In this case, the curve can be described by combining the two models as follows: the Z-Model to describe the first part of the curve until ε_IF1_, the F-Model for the second part of the curve, between ε_IF1_ and ε_IF2_, and finally the Z-Model for the last part of the curve. In the case of Figure 3BI the Z-Model has been used alone assuming the relation (2). The finding of the three constants has been done by means on least-squares method. In the case of Figure 3BII the F-Model used to describe the first part of the experimental data is (3) and a condition has been imposed to guarantee the continuity at TP. Such a condition is expressed by the following relation:

(15) $$B_{F}\,\left(e^{C_{F}\,\varepsilon_{\text{TP}}}-1\right)=\sigma_{\text{TP}}\Rightarrow B_{F}=\frac{\sigma_{\text{TP}}}{e^{C_{F}\,\varepsilon_{\text{TP}}}-1}$$

The Z-Model used to describe the second part of the experimental data is based on the relation (2) translated at the inflection point IF1 as follows:

(16) $$\sigma_{Z}\,\left(\varepsilon \right)=a_{Z}\,\left(\varepsilon -\varepsilon_{\mathrm{IF1}}\right)+b_{Z}\,\left[1-e^{-c_{Z}\,\left(\varepsilon -\varepsilon_{\mathrm{IF1}}\right)}\right]+\sigma_{\mathrm{IF1}}$$

The model fitting in the case of the data of Figure 3BIII has been done using an approach inspired by the procedure previously described (Tanaka et al., 2011; Lee et al., 2017). The first part of the curve (up to the IF1) in Figure 3BIII is described by the Z-Model in the form of relation (2). The second part of curve (up to the IF2) has been described by the F-Model translated in the first inflection point:

(17) $$\sigma_{F}\,\left(\varepsilon \right)=B_{F}\,\left[e^{C_{F}\,\left(\varepsilon -\varepsilon_{\mathrm{IF1}}\right)}-1\right]+\sigma_{\mathrm{IF1}}$$

The third part of the curve in Figure 3BIII has been described by the Z-Model translated at the second inflection point:

(18) $$\sigma_{Z}\,\left(\varepsilon \right)=a_{Z}\,\left(\varepsilon -\varepsilon_{\mathrm{IF2}}\right)+b_{Z}\,\left[1-e^{-c_{Z}\,\left(\varepsilon -\varepsilon_{\mathrm{IF2}}\right)}\right]+\sigma_{\mathrm{IF2}}$$

The joining conditions between the initial Z-Model and the F-Model which describe the second part of the data guarantee the physical continuity of the mechanical phenomenon. Meanwhile, to join the F-Model and the Z-model, used to fit the last part of the curve, a position continuity has been applied on a moving point starting from the second inflection point. By an iterative procedure, the slope of tangents of F-Model and Z-model are calculated in the moving point. The moving point at which the difference between slopes is minimum is considered as the transition point (see Figure 3C).

The best fitting of all models on experimental data have been done by means of the least-squares method.

## Results

### Thermal Characterization of PU

**FIGURE 4:**
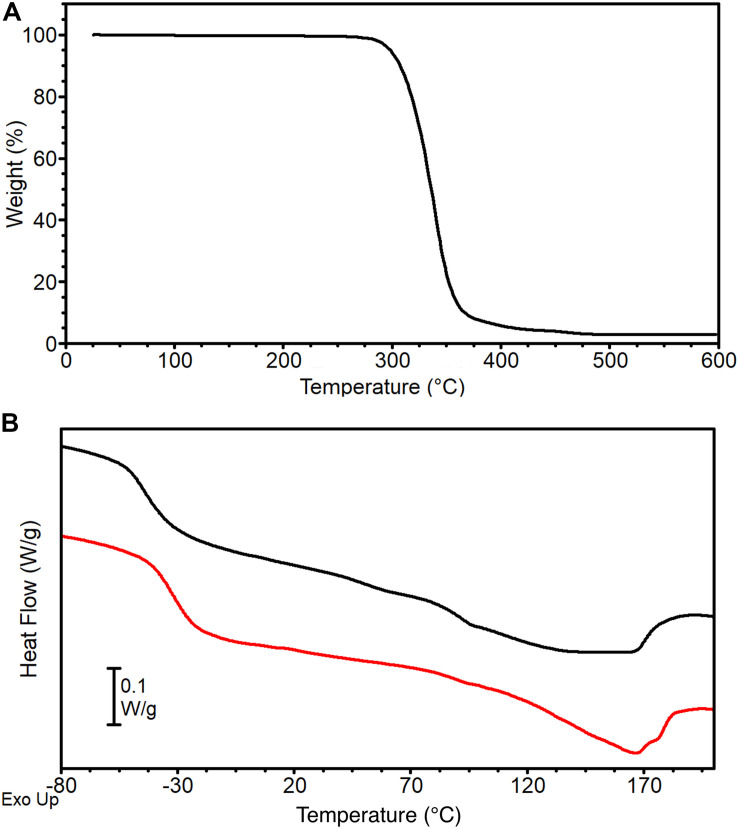
PU pellets thermal analysis: **(A)** TGA curve and **(B)** DSC 1st (black) and 2nd (red) heating scans.

TGA curve of PU pellet is reported in Figure 4A. Thermal degradation occurs in a single step in the temperature range 250–450°C, with a corresponding weight loss of about 96%, leading to an almost negligible residue of 4%. This result is in line with previous studies on thermoplastic MDI-based polyurethanes that report their low thermal stability (onset of degradation in the range 200–300°C) due to the cleavage of the urethane linkage to generate free-MDI, that further decomposes at higher temperatures into a mixture of nitrile compounds (such as HCN) and polyols residue, and to the breaking of ester bonds in the macroglycol groups (Herrera et al., 2002).

DSC curves of the PU pellets are reported in Figure 4B. The first heating scan (black curve) displays a T_g_ at −42°C followed by a broad endothermic peak with a maximum at 138°C (T_m_) and a corresponding melting endotherm ΔH_m_ of about 19 J g^–1^. The MDI-polyether-ester urethane used in the present work has a typical two-phase microstructure composed by hard and soft segments. Soft segments are formed by the polyether-ester moieties and have low glass transition temperature, while hard segments derived from the aromatic di-isocyanates and develop a crystal phase stabilized by H-bonds (Datta and Kasprzyk, 2018). The broadness of the melting endotherm can be ascribed to the different nature of H-bonds: NH groups of urethanes act as H-donor for both the urethane carbonyl and for the macroglycol group (ester carbonyl or ether oxygen). Peaks in the 70–80°C range have been ascribed to the disruption of urethane-macroglycol H-bonds at the interface of the microdomains, whereas peaks in the 150–180°C range to the breaking of urethane-urethane H-bonds (Seymour and Cooper, 1973). After cooling, the second heating scan (Figure 4B, red curve) shows a higher T_g_ compared to the first heating scan (T_g_ = −32°C), followed by a melting endotherm of lower entity (ΔH_m_ = 14 J g-^1^) that lacks the peak in the 70–80°C range typical of urethane-macroglycol H-bonds at microdomains interfaces, whereas its melting peak at T_m_ = 166°C suggests the presence of urethane-urethane H-bonds in the crystal phase. These differences can be explained by hypothesizing that the cooling has hindered phase segregation between soft and hard segments, thus decreasing the contribution of H-bond at the interphase whit an amorphous phase richer in MDI moieties.

### Morphology of the PU Structures

In order to reproduce the skeletal muscles myofibrils (Gillies and Lieber, 2011; Frontera and Ochala, 2015), electrospun mats of random and aligned PU nanofibers were compared. The SEM investigation revealed that both the random and aligned nanofibers were homogeneous, smooth, continuous, and with no defects such as beads.

**FIGURE 5:**
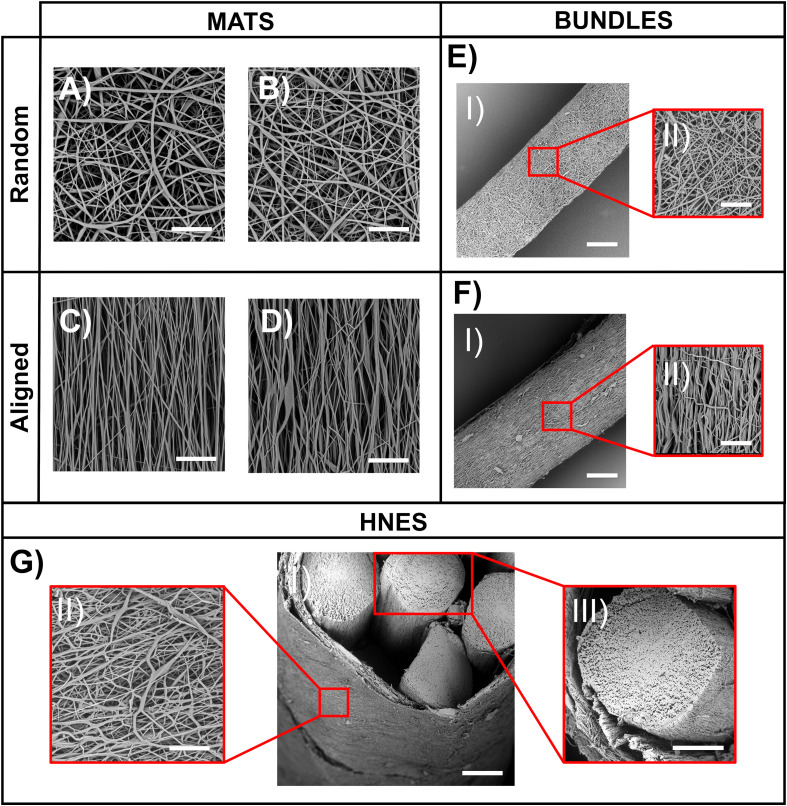
SEM analysis on electrospun specimens. **(A)** Mat of random fibers on PE support paper (scale bar = 20 μm); **(B)** Mat of random fibers removed from PE support paper (scale bar = 20 μm); **(C)** Mat of aligned fibers on PE support paper (scale bar = 20 μm); **(D)** Mat of aligned fibers removed from PE support paper (scale bar = 20 μm); **(EI)** Bundle of random fibers (scale bar = 100 μm); **(EII)** Bundle of random fibers – zoom in (scale bar 20 μm); **(FI)** Bundle of aligned fibers (scale bar = 100 μm); **(FII)** Bundle of aligned fibers – zoom in (scale bar 20 μm); **(GI)** HNES – Partial cross-section (scale bar = 150 μm); **(GII)** HNES – External membrane of random fibers (scale bar = 20 μm); **(GIII)** HNES – cross-section of one of the inner bundle of aligned fibers (scale bar = 100 μm).

The nanofibers of the random mats had a mean diameter of 0.95 ± 0.40 μm immediately after spun on the PE paper (Figure 5A), and of 0.89 ± 0.34 μm after detachment from the PE paper and a consequent shrinkage of about 3% of mat sides (Figure 5B). The aligned nanofibers showed a mean diameter of 0.65 ± 0.29 μm on the PE paper (Figure 5C) and 0.71 ± 0.33 μm after a shrinkage of about 11% of the length of the mat side parallel to fiber axis (Figure 5D) occurring upon paper detachment. The random mats had a thickness of 18.6 ± 2.3μm, and the aligned mats of 14.2 ± 1.4 μm. The volume fraction (*v*) for the random mats and of the aligned mats was 0.30 ± 0.03 and 0.37 ± 0.02, respectively.

To produce skeletal muscle fibers/fascicles inspired structures (Gillies and Lieber, 2011; Frontera and Ochala, 2015), ring bundles of random (Figure 5E) and axially aligned (Figure 5F) nanofibers were produced. The SEM investigation revealed that bundles were homogeneous (diameter of random bundles 468 ± 33 μm and aligned ones 419 ± 37 μm without the presence of beads (Figures 5EII,FII). After the removal from the drum collector, the aligned bundles showed a shrinkage of about 16%, while the random ones of about 3%. The volume fraction for the random bundles was 0.32 ± 0.02 and 0.44 ± 0.02 for the aligned bundles.

In order to resemble the whole structure of a skeletal muscle, as well as the epimysium membrane (Gillies and Lieber, 2011; Frontera and Ochala, 2015), hierarchical nanofibrous electrospun structures (HNES) were produced by grouping ring bundles of aligned nanofibers (Figure 5G). The electrospun membrane tightened and strongly packed the internal bundles resulting in the final HNES, showing a cylindrical shape with a diameter of 1.14 ± 0.17 mm. The SEM investigation showed that the random nanofibers of the membrane (Figure 5GII) were smooth, continuous, and homogeneous with a mean diameter of 0.88 ± 0.36 μm. The volume fraction was 0.47 ± 0.08.

### Orientation of the Nanofibers in the PU Structures

**FIGURE 6:**
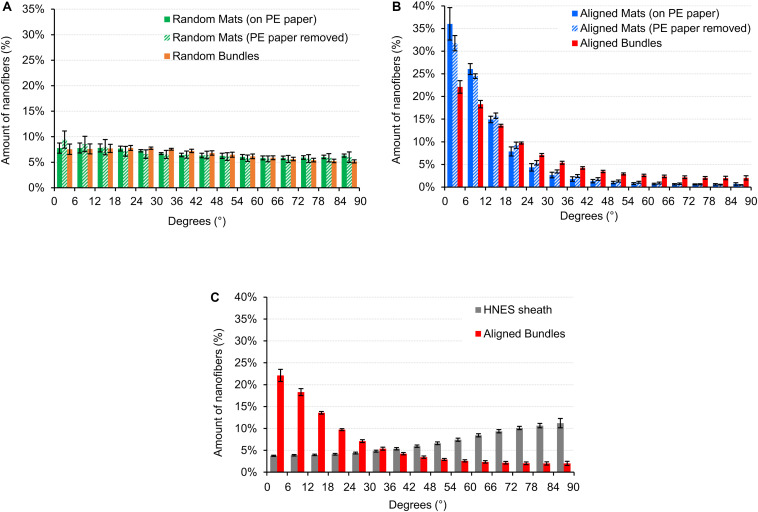
Directionality of the nanofibers in the random and aligned specimens and on the HNES membrane. An angle of 0° corresponds to the longer axis of the specimen, an angle of 90° corresponds to the perpendicular direction. The directionality histograms show the distribution of the nanofibers in the different directions for: **(A)** Mats (before and after the PE paper removal) and bundles of random fibers; **(B)** Mats (before and after the PE paper removal) and bundles of aligned fibers; **(C)** HNES external membrane compared with the aligned bundles.

The directionality analysis carried out on the mats and bundles of random fibers is showed in Figure 6A. As expected, no preferential orientation of the fibers in one specific direction was detected, both before and after PE paper removal.

The mats and bundles of aligned fibers showed a preferential axial orientation of the nanofibers with a progressive circumferential scatter (Figure 6B). For the mats (both before and after PE paper removal) the amount of nanofibers oriented in the range 0–18° was about 75% while fewer than 3% were in the range 72–90°. Samples on PE paper showed a slightly greater alignment. Similarly, in the bundles the percentage of nanofibers oriented in the range 0–18° was 54% and in the range 72–90° was of 8.1%.

The nanofibers in the HNES membrane instead, revealed a slightly circumferential orientation with an amount of nanofibers of 41.3% in the range 72–90° (Figure 6C).

### Mechanical Properties of the PU Structures

**FIGURE 7:**
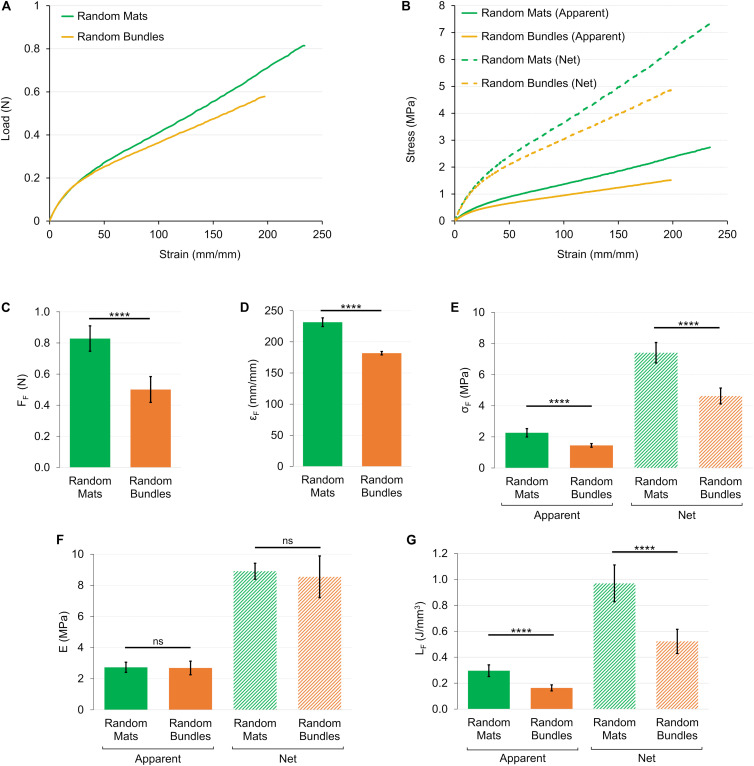
Mechanical tensile characterization of the mats and bundles of random fibers. **(A)** Representative load-strain curves. **(B)** Representative apparent (solid lines) and net (dashed lines) stress–strain curves. Comparison between the mechanical properties of mats and bundles of random fibers: **(C)** Failure force (F_F_); **(D)** Failure strain (ε_F_); **(E)** Failure stress (σ_F_) (apparent = solid bars; net = dashed bars); **(F)** Elastic Modulus (E) (apparent = solid bars; net = dashed bars); **(G)** Unit work to failure (L_F_) (apparent = solid bars; net = dashed bars). The mean and standard deviation are represented for each group. The statistical significance of differences is indicated (****p < 0.0001, ns, not significant).

The load-strain curves of the mats and bundles of random fibers revealed a similar ductile behavior with large deformations without a non-linear toe region (Figure 7A). The random mats had a failure force of F_F_ = 0.83 ± 0.08 N (ε_F_ = 232 ± 17%), while for bundles of F_F_ = 0.50 ± 0.08 N (ε_F_ = 182 ± 18%) (Figures 7C,D). The elastic region (random mats E_A_ = 2.73 ± 0.33 MPa; random bundles E_A_ = 2.69 ± 0.44 MPa) ended at an apparent yield stress of σ_AY_ = 0.23 ± 0.03 MPa (ε_Y_ = 8.23 ± 1.06%) for the random mats and of σ_AY_ = 0.22 ± 0.05 MPa (ε_Y_ = 7.54 ± 1.90%) for the random bundles (Figure 7F). Both the random structures showed a ductile region up to an apparent failure stress of σ_AF_ = 2.27 ± 0.27 MPa for the mats and of σ_AF_ = 1.45 ± 0.12 MPa for the bundles (Figure 7E). After the failure stress, both types of samples showed an immediate break, reaching a unit work to failure of L_AF_ = 0.30 ± 0.05 J/mm^3^ for the random mats and of L_AF_ = 0.16 ± 0.02 J/mm^3^ for the random bundles (Figure 7G). The net mechanical properties were about three times as high as the apparent ones for both type of random samples (Figure 7B and Table 1). For the detailed statistical significances, see Supplementary Material.

**TABLE 1:** Apparent and Net mechanical properties for the mats, bundles and HNES.

		Random Mats	Random Bundles	Aligned Mats	Aligned Bundles	HNES
F_AF_	(N)	0.83 ± 0.08	0.50 ± 0.08	3.90 ± 0.59	6.70 ± 0.95	19.1 ± 3.8
σ_A__Y_	(MPa)	0.23 ± 0.03	0.22 ± 0.05	5.26 ± 0.27	6.69 ± 0.64	8.45 ± 0.47
σ_A__F_	(MPa)	2.27 ± 0.27	1.50 ± 0.12	13.8 ± 1.5	24.0 ± 1.4	18.9 ± 1.7
ε_Y_	(%)	8.23 ± 1.06	7.54 ± 1.90	14.6 ± 0.6	29.6 ± 1.5	46.0 ± 1.3
ε_F_	(%)	232 ± 17	182 ± 18	48.9 ± 7.0	104 ± 3	100 ± 1
E_A_	(MPa)	2.70 ± 0.33	2.70 ± 0.44	59.8 ± 2.5	37.9 ± 2.3	30.5 ± 1.8
AS_A_	(MPa)	0.87 ± 0.09	0.65 ± 0.05	25.5 ± 2.4	23.5 ± 1.6	19.5 ± 1.9
L_AY_	(J/mm^3^)	0.0011 ± 0.0003	0.0010 ± 0.0004	0.028 ± 0.002	0.082 ± 0.007	0.15 ± 0.01
L_AF_	(J/mm^3^)	0.30 ± 0.05	0.16 ± 0.02	0.38 ± 0.10	1.27 ± 0.08	0.92 ± 0.07
σ_N__Y_	(MPa)	0.71 ± 0.08	0.80 ± 0.15	12.6 ± 0.5	15.1 ± 0.7	18.0 ± 2.0
σ_N__F_	(MPa)	7.41 ± 0.66	4.63 ± 0.51	33.0 ± 3.5	54.7 ± 1.4	40.2 ± 3.2
E_N_	(MPa)	8.90 ± 0.51	8.55 ± 1.34	141 ± 3	86.2 ± 2.6	65.1 ± 7.0
AS_N_	(MPa)	2.83 ± 0.11	2.06 ± 0.15	61.0 ± 6.1	53.6 ± 1.4	41.5 ± 3.3
L_NY_	(J/mm^3^)	0.0035 ± 0.0009	0.0030 ± 0.0014	0.067 ± 0.005	0.19 ± 0.01	0.33 ± 0.04
L_NF_	(J/mm^3^)	0.97 ± 0.14	0.52 ± 0.09	0.92 ± 0.24	2.90 ± 0.15	1.95 ± 0.20

**FIGURE 8:**
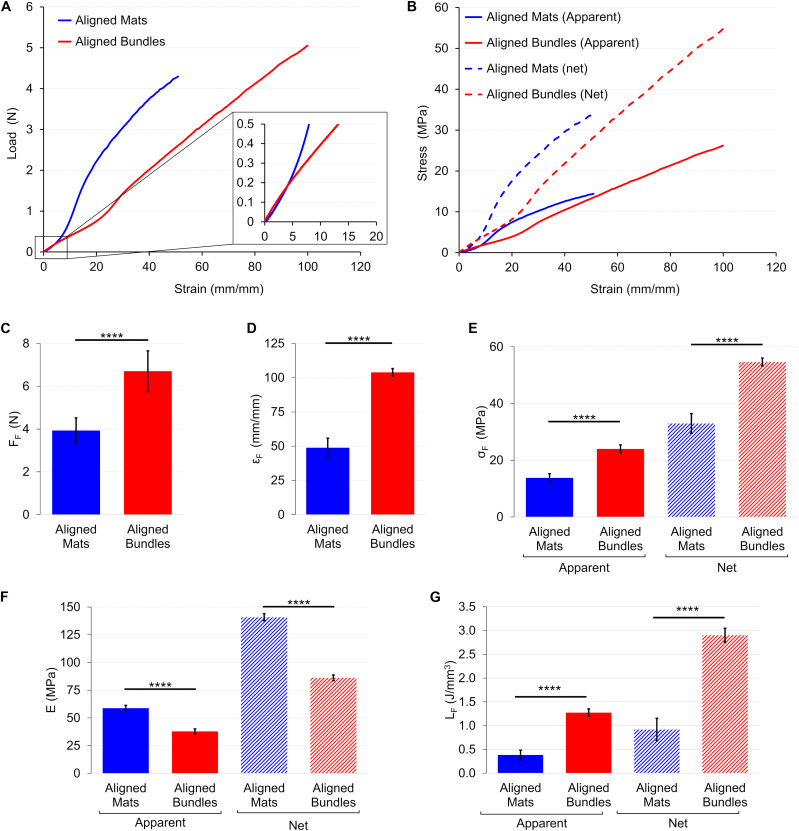
Mechanical tensile characterization of the mats and bundles of aligned fibers. **(A)** Typical load-strain curves. **(B)** Typical apparent (solid lines) and net (dashed lines) stress–strain curves. Comparison between the mechanical properties of mats and bundles of aligned fibers: **(C)** Failure force (F_F_); **(D)** Failure strain (ε_F_); **(E)** Failure stress (σ_F_) (apparent = solid bars; net = dashed bars); **(F)** Elastic Modulus (E) (apparent = solid bars; net = dashed bars); **(G)** Unit work to failure (L_F_) (apparent = solid bars; net = dashed bars). The mean and standard deviation are represented for each group. The statistical significance of differences is indicated (****p < 0.0001).

The load-strain curves of mats and bundles of aligned fibers showed a ductile behavior with a non-linear toe region (Figure 8A). In particular, the toe region of the aligned bundles was ∼2.5 times wider than that of the mat ones. The mats had a failure force of F_F_ = 3.93 ± 0.59 N (ε_F_ = 48.9 ± 7.0%), while the bundles of F_F_ = 6.71 ± 0.95 N (ε_F_ = 104 ± 3%) (Figures 8C,D). The elastic region for the mats was clearly distinguishable with an apparent elastic modulus of E_A_ = 58.9 ± 2.5 MPa. The bundles instead, showed a short linear region with an apparent elastic modulus of E_A_ = 37.9 ± 2.3 MPa (Figure 8F). The yield of the mats occurred at an apparent yield stress of σ_AY_ = 5.26 ± 0.27 MPa (ε_Y_ = 14.6 ± 0.6%) while for the bundles of σ_AY_ = 6.69 ± 0.64 MPa (ε_Y_ = 29.6 ± 1.5%). Both the structures of aligned fibers showed a ductile region up to an apparent failure stress of σ_AF_ = 13.8 ± 1.5 MPa for the mats and of σ_AF_ = 24 ± 1 MPa for the bundles (Figure 8E). The unit work to failure was L_AF_ = 0.38 ± 0.10 J/mm^3^ for the aligned mats and L_AF_ = 1.27 ± 0.08 J/mm^3^ for the Aligned bundles (Figure 8G). After the failure stress, the bundles showed an immediate break, while the mats progressively frayed from the sides to the center of the specimens until a complete detachment. For both the mats and the bundles of aligned fibers the net mechanical properties were about 2.5 times as high as the apparent ones (Figure 8B and Table 1). For the detailed statistical significances, see Supplementary Material.

**FIGURE 9:**
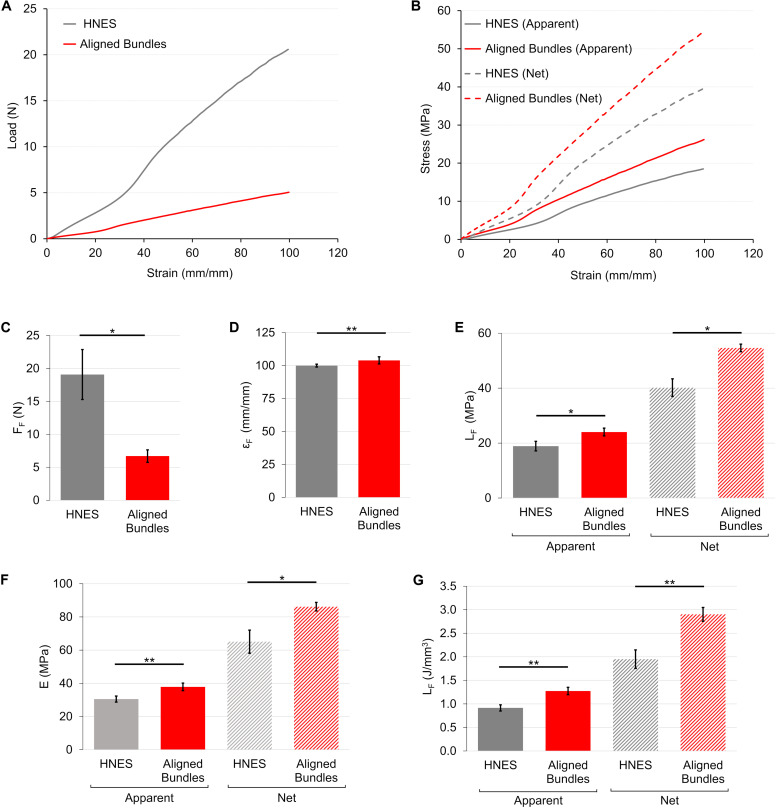
Mechanical tensile characterization of the bundles of aligned fibers and of HNES. **(A)** Representative load-strain curves. **(B)** Representative apparent (solid lines) and net (dashed lines) stress–strain curves. Comparison between the mechanical properties of aligned bundles and HNES: **(C)** Failure force (F_F_); **(D)** Failure strain (ε_F_); **(E)** Failure stress (σ_F_) (apparent = solid bars; net = dashed bars); **(F)** Elastic Modulus (E) (apparent = solid bars; net = dashed bars); **(G)** Unit work to failure (L_F_) (apparent = solid bars; net = dashed bars). The mean and standard deviation are represented for each group. The statistical significance of differences is indicated (*p ≤ 0.05, **p ≤ 0.01).

The load-strain curve for the HNES showed a ductile behavior with a large toe region up to strain value of ∼40%, two times wider than the aligned bundles (Figure 9A). The break of the specimens occurred at failure force of F_F_ = 19.1 ± 3.8 N (ε_F_ = 100 ± 1%) (Figures 9C,D). After a well-defined elastic region (E_A_ = 30.5 ± 1.8 MPa) (Figure 9F) the HNES started to yield at an apparent yield stress of σ_AY_ = 8.45 ± 0.47 MPa (ε_Y_ = 46.0 ± 1.3%). The apparent failure stress was σ_AF_ = 18.9 ± 1.7 MPa with a progressive break of the bundles (Figure 9E). The unit work to failure was LAF = 0.92 ± 0.07 J/mm^3^ (Figure 9G). The net mechanical properties were about twice as high as the apparent ones (Table 1). For the detailed statistical significances, see Supplementary Material.

### Model Fitting of the Mechanical Behavior of the PU Structures

**FIGURE 10:**
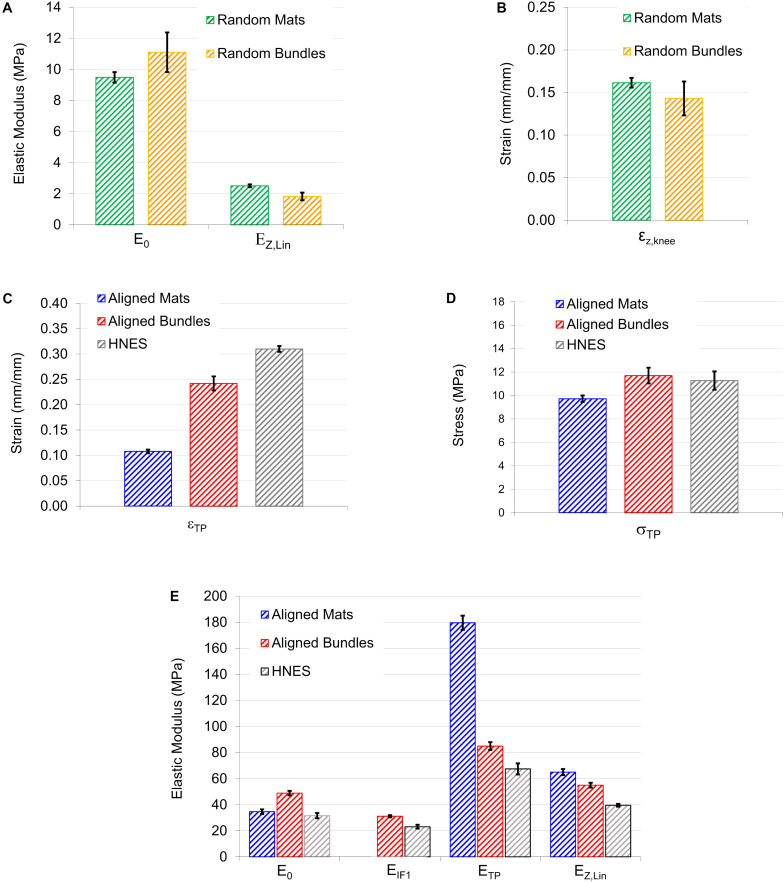
Graphical summary of the most relevant mechanical information calculated from models: **(A,B)** Show the elastic moduli and the strains at knee obtained by Z-Model fitted on samples with randomly oriented nanofibers; **(C,D)** are, respectively, the strain and the stress of the transition point obtained by the iterative procedure; **(E)** Elastic moduli calculated at the four main position on the stress-strain curves. In particular E_0_ of aligned mats was calculated by F-Model on the toe region; E_0_ of aligned bundles and HNES were calculated by Z-Model fitted on the initial region; E_TP_ was calculated as mean of the values calculated by the F-Model and Z-Model in TP.

Models successfully fitted the experimental net stress-strain curves for all the different sample families. The parameters resulted from the Z-Model fitting of the random mats and bundles are listed in Table 2 and Figures 10A,B.

**TABLE 2:** Mechanical parameters for the random mats and bundles obtained by the Z-model.

		Randommat	Randombundle
a_Z_	(MPa)	2.51 ± 0.09	1.82 ± 0.24
b_Z_	(MPa)	1.13 ± 0.06	1.35 ± 0.16
c_Z_	(–)	6.20 ± 0.28	6.93 ± 1.08
E_Z,0_	(MPa)	9.49 ± 0.34	11.1 ± 1.9
E_Z,Lin_	(MPa)	2.51 ± 0.09	1.82 ± 0.24
ε_Z,knee_	(mm/mm)	0.16 ± 0.01	0.14 ± 0.02
Δ_Z_	(%)	−73.5	−83.5

For the random mats, the initial elastic modulus E_0_ was 9.49 ± 0.34 MPa while for the random bundles was 11.1 ± 1.9 MPa. After the non-linear region, where the material exhibits softening due to relaxation of entanglements (Maccaferri et al., 2019), the linear elastic asymptotic modulus E_Z,Lin_ (which is theoretically similar to the AS) for the random mats was 2.51 ± 0.09 MPa, while for the random bundles was 1.82 ± 0.24 MPa. In the case of random mats, the percentage stiffness variation between E_0_ and E_Z,Lin_ was Δ_Z_ = -73.5%, while, for the random bundles it was Δ_Z_ = -83.5%. The strain of the knee point, ε_Z,knee_, for the random mats, was 0.16 ± 0.01 while for the random bundles of 0.14 ± 0.02.

The parameters resulted from the model fitting of the aligned mats, bundles and HNES are listed in Table 3 and Figures 10C–E.

**TABLE 3:** Mechanical parameters for the aligned mats, bundles and HNES obtained by the model fitting.

		Alignedmat	Alignedbundle	HNES
ε_IF1_	(mm/mm)	–	0.01 ± 0.02	0.10 ± 0.017
σ_IF1_	(MPa)	–	3.82 ± 0.61	2.84 ± 0.47
ε_TP_	(mm/mm)	0.104 ± 0.004	0.24 ± 0.01	0.31 ± 0.07
σ_TP_	(MPa)	9.21 ± 0.23	11.7 ± 0.7	11.27 ± 0.79
ε_IF2_	(mm/mm)	0.108 ± 0.003	0.25 ± 0.01	0.31 ± 0.01
σ_IF2_	(MPa)	9.71 ± 0.28	12.3 ± 0.6	11.4 ± 0.86
a_Z,i_	(MPa)	–	34.8 ± 1.0	25.6 ± 0.4
b_Z,i_	(MPa)	–	0.58 ± 0.12	0.29 ± 0.08
c_Z,i_	–	–	25.1 ± 3.7	28.1 ± 3.4
B_F_	(MPa)	2.20 ± 0.12	4.55 ± 0.26	4.65 ± 0.90
C_F_	–	15.8 ± 0.5	6.87 ± 0.46	5.06 ± 0.75
a_Z,f_	(MPa)	65.0 ± 2.3	55.0 ± 1.9	39.6 ± 1.1
b_Z,f_	(MPa)	5.98 ± 0.47	2.25 ± 0.28	2.87 ± 0.69
c_Z,f_	–	19.3 ± 1.7	13.6 ± 2.6	9.87 ± 1.35
E_0_	(MPa)	34.6 ± 1.8**	48.8 ± 1.7*	31.6 ± 2.1*
E_IF1_	(MPa)	–	31.1 ± 0.8	23.1 ± 1.4
E_TP_	(MPa)	179.5 ± 5.4	85.0 ± 3.0	67.4 ± 4.2
E_Z,Lin_	(MPa)	65.0 ± 2.3	55.0 ± 1.9	39.6 ± 1.1
ε_Z,knee,i_	(mm/mm)	–	0.040 ± 0.005	0.036 ± 0.004
ε_Z,knee,f_	(mm/mm)	0.052 ± 0.004	0.07 ± 0.01	0.10 ± 0.01
Δ_Z,i_	(%)	–	−26	−27
Δ_F_	(%)	420	173	193
Δ_Z,f_	(%)	-64	−35	−41

* Calculated on the basis of Z-Model, E_*F,0.*_ ** Calculated on the basis of F-Model, E_*Z,0.*_

The values of strain at the first inflection point (ε_IF1_) were, for the aligned bundles 0.01 ± 0.02 and for the HNES 0.10 ± 0.017, with a mean percentage difference of 90% between them. The stress at the first inflection point (σ_IF1_) instead was 3.82 ± 0.61 MPa for the bundles and 2.84 ± 0.47 MPa for the HNES, with a mean percentage difference of 26%. Moreover, for aligned bundles and HNES, it is possible to note that the strain at the knee, in the initial Z-Model region, is similar: for the bundles ε_Z,knee,i_ was 0.040 ± 0.005 and in the case of HNES it was 0.036 ± 0.004. From this analysis it is relevant to notice that bundles and HNES are characterized by the same knee strain.

It should be noted that all structures with aligned nanofibers have similar, though not equal, initial stiffness values. However, in the case of aligned mats, this initial stiffness value was higher, as well described by the F-Model applied to the initial part of the stress-strain curve. Reversely, in the case of bundles and HNES, the initial stiffness value was lower as predicted by the Z-Model which well describes the softening occurring in their early stages of the stress-strain curve. In particular, it was observed that for the aligned mats the initial modulus, E_0_ = 48.8 ± 1.7 MPa, decreased down to E_IF1_ = 31.1 ± 0.8 MPa with a stiffness variation of Δ_Z,i_ = –26%. While, in the case of HNES the initial modulus was a bit less respect to the mats, E_0_ = 31.6 ± 2.1 MPa and it decreased down to E_IF1_ = 23.1 ± 1.4 MPa at the first inflection point, with a stiffness variation of Δ_Z,i_ = -27%. From this analysis it is evident that even if the initial modulus of the bundles and HNES are comparable but not equal (48.8 MPa vs. 31.1 MPa), their variation until the inflection point is equal (26 vs. 27%).

The aligned specimens showed incremental values of the strain at TP from the mats up to the HNES (mats ε_TP_ = 0.108 ± 0.003; bundles ε_TP_ = 0.24 ± 0.01; HNES ε_TP_ = 0.31 ± 0.07) while maintaining approximately the same mean values of stress at the TP (mats σ_TP_ = 9.72 ± 0.28 MPa; bundles σ_TP_ = 11.7 ± 0.7 MPa; HNES σ_TP_ = 11.27 ± 0.79 MPa) (Figures 10C,D).

In the final region of the curves described by the Z-Model, the ε_Z,knee,f_ for the mats was 0.052 ± 0.004%, for the bundles was 0.070 ± 0.01% and for the HNES was 0.100 ± 0.01%. The differences of mean values of the ε_Z,knee,f_ in the three types of samples progressively increased, passing from the mats up to the HNES. In particular, for the mats and the bundles the difference in ε_Z,knee,f_ was 26%, while in the case of aligned bundles and HNES such a difference was about 30%.

The specimens with aligned nanofibers showed also a reduction for all calculated E_TP_ and E_Z,Lin_ due to their increased structural complexities, ranging from the mats to the HNES (Figure 10E). After the end of the non-linear toe region of diagrams, for all aligned samples a relevant increment in the elastic moduli at the TP happened in the region described by the F-Model. In particular, in the case of aligned mats the stiffness value at the initial point of the F-Model was 34.6 ± 1.8 Mpa, while at the end of the F-Model, in the TP point, the stiffness increased up to 179.5 ± 5.4 MPa, with a huge percentage stiffness variation of Δ_F_ = 420%. In the case of aligned bundles, the initial value of the stiffness for the F-Model region was 31.1 ± 0.8 MPa and it increased up to 85.0 ± 3.0 MPa at the TP, with a percentage increment of Δ_F_ = 173%. Finally, in the case of HNES, the stiffness increased in the F-Model region from 23.1 ± 1.4 MPa up to 67.4 ± 4.2 MPa, with an average percentage increment of Δ_F_ = 193%.

For all samples with aligned nanofibers the Z-Model applied to the last part of the curve enabled the calculation of the linear-asymptotic elastic modulus, and in particular for the mats E_Z,Lin_ was 65.0 ± 2.3 MPa, for the bundles it was 55.0 ± 1.9 MPa and for the HNES it was 39.6 ± 1.1 MPa. These values have given evidence of a strain-softening if compared to stiffness results obtained by the F-Model region. In particular, by comparing the values of E_Z,Lin_ to the E_TP_ it was possible to calculate the softening effect in terms of stiffness reduction as follows: for the aligned mats Δ_Z,f_ = -64%, for the aligned bundles the variation was Δ_Z,f_ = -35% and for the HNES such a variation was Δ_Z,f_ = -41%.

## Discussion

**FIGURE 11:**
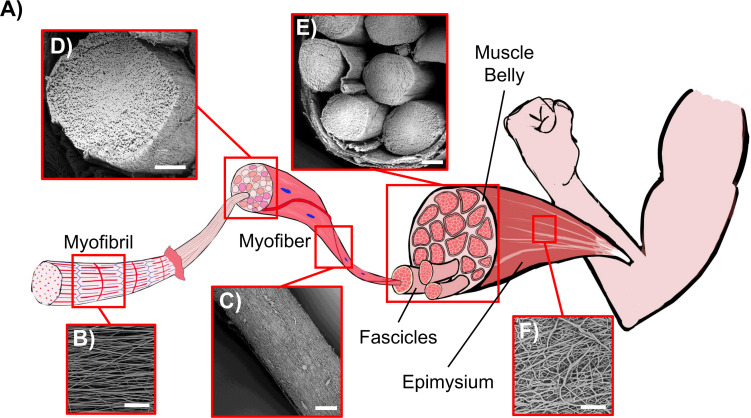
Comparison between the biological skeletal muscle and the electrospun PU structures. **(A)** Schematics of the hierarchical structure of the skeletal muscle. **(B)** Mat of aligned nanofibers. The single nanofiber corresponds to a myofibril (scale bar = 20 μm); **(C)** Bundle of aligned fibers, corresponding to a myofiber (scale bar = 100 μm); **(D)** Cross-section of an aligned bundle, showing the parallel arrangement of the inner nanofibers (scale bar = 100 μm). **(E)** Cross-section of the HNES, compared to the cross-section of a biological muscle belly (scale bar = 150 μm); **(F)** HNES membrane, resembling the epimysium membrane that envelops the muscle belly (scale bar = 100 μm).

One of the greatest challenges of biomimicry is to reproduce the complex fibrous morphology and performances of skeletal muscles. In this work an innovative hierarchical electrospun nanofibrous structure was successfully developed, mimicking its biological counterpart with a bottom-up approach (Figure 11). A thermoplastic PU was chosen as passive material to produce the muscle-like structure. This class of polymers is characterized by a two-phase microstructure composed of hard and soft segments. By varying the ratio, the molecular weight and the chemical structure of the hard and the soft segments it is possible to largely modulate polymer mechanical properties, in terms of elastic modulus, stress and strain at failure and toughness, thus achieving mechanical behaviors that span from that of soft elastomers to that of hard plastics (Datta and Kasprzyk, 2018). The PU used in the present work contains polyether-esters soft segments with very low T_g_ that can confer high deformability and low elastic modulus, making it suitable to act as passive material for artificial muscles. At the same time the chosen polymer contains aromatic di-isocyanate units (MDI) able to crystallize even during fast cooling (in DSC, Figure 4B) and fast fiber solidification occurring during electrospinning, a crucial characteristic to allow the maintenance of fiber morphology at room temperature.

By optimizing the electrospinning parameters, PU nanofibers with a diameter in the same order of magnitude of the skeletal muscle myofibrils (about 1 μm) (Fung, 1993) were obtained with a random orientation (Figures 5A,B). Subsequently, considering the well-known axial alignment of skeletal muscle myofibrils (Frontera and Ochala, 2015), the aligned mats were also produced to increase the morphological biomimicry (Figure 4C). Interestingly, after the removal of the PE support paper, the nanofibers assumed a slightly crimped arrangement (resulting in mat shrinkage) (Figure 5D), that has the effect to increase the non-linear toe region recorded during tensile tests, typical of many soft tissues, including skeletal muscles (Black and Hastings, 2016). Crimping is evident, for instance, in the relaxed state of tendons collagen fibers; conversely is less visible in the skeletal muscle tissue due to the muscle tone. The shrinkage of electrospun fibers has been ascribed to changes of molecular conformation from an extended to a relaxed and more entropically stable one. Extended chain conformations are generated during electrospinning under the action of the electrical field (Richard-Lacroix and Pellerin, 2013). These extended conformations persist until fibers are kept under tension, adherent to the PE paper, and relaxed after detaching, with a consequent macroscopic shrinkage. When collected on drum rotating at high speed, chains can be further stretched (Edwards et al., 2010; Mohan et al., 2011), thus displaying a higher degree of shrinkage upon relaxation and a longer non-linear toe region when tensioned (Surrao et al., 2010).

To scale up the hierarchical arrangement, bundles of nanofibers were produced by rolling up the mats to mimic the muscle fibers (Figures 5E,F). By tuning the width of the starting mat, it was possible to obtain bundle diameters in the same order of magnitude of those of muscle fibers (about 100 μm) and fascicles (up to 280 μm) (Shahinpoor et al., 2007; Frontera and Ochala, 2015; Ceylan et al., 2017; Klećkowska-Nawrot et al., 2018). To define the best compromise between morphology and mechanical performances, both random and aligned bundles were investigated. After the rolling up procedure, the aligned bundles maintained an evident axial alignment of the nanofibers, confirmed by the Directionality analysis carried out on SEM images (Figure 6B). This aligned arrangement better resembles the parallel packs of myofibrils inside the muscle fibers with respect to the bundles of random ones (Figures 11B–D). In addition, the high shrinkage experienced by the aligned bundles induced a crimping of the nanofibers (Figure 5FII), while maintaining their preferential axial alignment, that can generate a non-linear elastic behavior, typical of muscle tissue.

To further emulate the complex structure of native muscle tissue, where skeletal muscle fibers and fascicles are surrounded by randomly arranged collagen-rich fibrous membranes (epimysium and perimysium), an external electrospun PU nanofibrous membrane was used. This membrane grouped and tightened a bunch of axially aligned bundles (Figure 5GII), obtaining a HNES structurally comparable to the whole skeletal muscle tissue (Gillies and Lieber, 2011). Consistently with the natural one, this membrane was produced with a random pattern of the nanofibers (Figures 5GII, 11F). Moreover, a slight circumferential preferential alignment of the fibers was demonstrated (Figure 6C), conferring an enhanced tightening of the group of internal bundles.

To achieve a good replication of the native muscle, the 3D hierarchical morphology should be combined with biomimetic mechanical properties. A systematic mechanical characterization of all the investigated samples, from mats to bundles and finally the HNES, was performed. When random fibers were used, the mechanical properties of mats and bundles had a similar stress-strain pattern (Figure 7A). After a first overlapping linear region, the bundles showed a lower asymptotic stiffness compared to the mats, as previously observed (Pauly et al., 2016; Sensini et al., 2019a). The obtained curves are similar to those commonly reported in the literature for random electrospun structures (Ridruejo et al., 2011; Molnar et al., 2012; Sinha-Ray et al., 2014). In detail, a small linear region (up to about 8% strain), followed by a large ductile asymptotic trend (at a stiffness of about 0.7 MPa) was observed until failure (230% strain for mats and 182% for bundles). Some of the apparent mechanical values (i.e., mats: E_A_ = 2.73 ± 0.33 MPa, σ_AF_ = 2.27 ± 0.27 MPa; bundles: E_A_ = 2.69 ± 0.44 MPa, σ_AF_ = 1.45 ± 0.12 MPa) are in the same range of the biological counterpart (muscle fibers: E_A_ = 0.02–1.6 MPa; σ_AF_ up to 2 MPa) (Moss and Halpern, 1977; Wolff et al., 2006; Kuthe and Uddanwadiker, 2016), while the strain levels at failure are considerably higher (muscle myofibrils and fibers: 30–60%). However, the curves did not show the toe region as well as the progressive stiffening which is typical of skeletal muscle (Herbert, 1988; Full and Meijer, 2000; Toursel et al., 2002; Takaza et al., 2012).

Differently, mats and bundles of aligned fibers have a more biomimetic mechanical behavior since they display a non-linear trend characterized by a toe region (up to about 10% for the mat and 20% for the bundle) (Figures 8A,B). The length of the toe region was unexpectedly more extended in the bundles than in the mats (Figures 8A,B), probably as a consequence of the higher degree of alignment of nanofibers in the mats (about 75% in the range 0–18°) compared to that found in the bundles (only 54% in the range 0–18°) (Figure 6). The lower degree of fiber alignment in bundles causes a delayed straightening and a reduction of the overall stiffness (E_A_ = 37.9 ± 2.3 MPa) with respect to the mats (E_A_ = 58.9 ± 2.5 MPa), both higher than the muscle fiber one (E_A_ = 0.02–1.6 MPa). After yielding the bundles failed at ε_F_ = 104 ± 3%, showing a failure deformation twice as high as that of the mat (ε_F_ = 48.9 ± 6.99%), and comparable to that of the skeletal muscles fibers (30–60%) (Kovanen et al., 1984; Hoang et al., 2007; Kuthe and Uddanwadiker, 2016; Schleifenbaum et al., 2016). The apparent failure stress of the bundle was σ_AF_ = 24.0 ± 1.4 MPa and 13.8 ± 1.5 MPa for the mat, higher than the muscle fiber one (σ_AF_ up to 2 MPa) (Kuthe and Uddanwadiker, 2016).

The HNES displays a mechanical behavior similar to that of the single bundles but with increased value of strain at the end of the toe region (about 35% of HNES compared to 20% of aligned bundle). The elastic modulus of the HNES (E_A_ = 30.5 ± 1.8 MPa) was also slightly lower than that of aligned bundles (E_A_ = 37.9 ± 2.3 MPa), thus contributing to approach the target value of the biological muscle (E_A_ up to 8 MPa). The external membrane grouped and tightened the bunch of bundles until failure which occurred near the capstan grips, probably caused by a stress concentration. This produced a reduction of ∼29% from the estimated HNES failure force, calculated as the sum of the mean failure force of its inner bundles.

In general, the values of net stresses, calculated considering the volume fraction, were higher compared to the apparent ones (Figures 7B, 8B, 9B) that are calculated considering a volume overestimated due to porosity.

The introduction of models enabled a deeper investigation of the mechanical behavior of the nanofibrous materials and in particular, their engineering perspective application in soft robotics and *in silico*-modeling of biomimetic structures. The here adopted modeling approach enabled the analysis of parts of stress-strain curves which have not been studied in researches which consider biological fibrous soft tissues (Fung, 1967; Vladimir and Mow, 1980; Tanaka et al., 2011; Goh et al., 2014; Lee et al., 2017) and, moreover, it allowed to highlight some intermediate phenomena, as the knee points, which were never considered before.

In the case of randomly oriented nanofibrous materials, it was possible to notice that in both geometrical arrangements, mats and bundles, they exhibited a similar mechanical behavior up to the strain knee with almost identical values (for the mats ε_Z,knee,I_ = 0.16 and for the bundles ε_Z,knee,I_ = 0.14, see Figure 10B and Table 2). Then the bundle assembling of the nanofibrous material exhibited a less stiff behavior for large strain values and was more prone to damage, as highlighted by the global stiffness variation (Δ_Z_ = −74% in the case of mats and Δ_Z_ = -84% in the case of bundles), with respect to the mats.

The analysis of aligned mats and HNES revealed that the early stage of stress-strain curves were characterized by a softening behavior, before the toe region, which exhibited a measurable stiffness reduction (from E_0_ to E_IF1_ the variation Δ_Z,i_ was about 26%) in a limited strain domain (ε_IF1_ was about 0.1). Additionally, both aligned mats and HNES were characterized by very similar values of strain at knee. This means that for both structures the transition from non-linear to linear dominated behavior happened at the same strain level (ε_Z,knee_ was about 0.038). From the analysis of the stress-strain curve in the pre-toe region it appears that both bundles and HNES exhibited a very similar behavior which could be physically related to frictional phenomena that influence the sliding between nanofibers.

**FIGURE 12:**
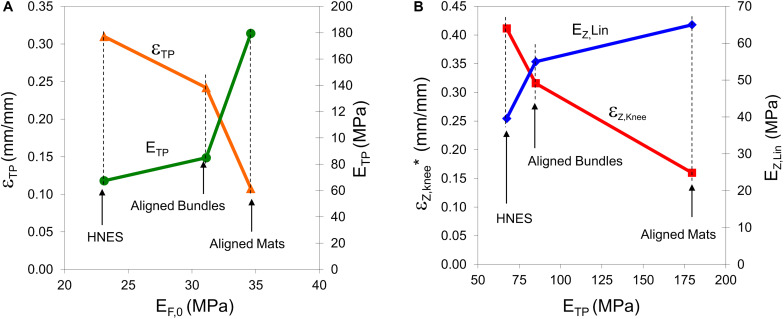
**(A)** Strain at the transition point (ε_TP_), and elastic modulus at the transition point (E_TP_) vs. the elastic modulus at the initial point of the toe region (E_F,0_, see Table 3) in the case of aligned samples. **(B)** Strain at the knee point (ε_Z,knee_* = ε_Z,knee_+ε_TP_) and linear asymptotic modulus (E_Z,lin_) vs. the elastic modulus at the transition point (E_TP_) in the case of aligned samples.

The toe region showed a stiffening behavior of the material which was well described by the F-Model and which was significantly influenced by the geometrical arrangements of the aligned nanofibers. This region has been characterized by three mechanical features: the initial elastic modulus (E_Z,__0_), the position of the transition point (ε_TP_) and the elastic modulus at the transition point (E_TP_). In Figure 12A it is possible to note that the greater is the initial elastic modulus the lesser is the strain of the transition point and the greater is the elastic modulus at the transition point. The initial elastic modulus is representative of the initial stiffness of the material, and it is related to the material attitude to react against the imposed deformation. In the case of the aligned mats, the high value of the initial elastic modulus is due to a high geometrical order of the nanofibers even if they were shrunk. On the contrary, in the case of bundles and HNES, which is an assembly of bundles, the order of the aligned nanofibers can be partially lost because of the manufacturing process and in particular due to the rolling up procedure (see Figure 5). Thus, the initial elastic modulus of bundles and HNES is lower with respect the that of the aligned mats. Moreover, the lower is the initial elastic modulus, the greater has to be the strain required to reorder the nanofibers and to recover their shrinkage (see Figure 12A). In particular, in the case of mats the degree of nanofibers alignment is high (about 75% of the nanofibers are in the range 0–18 degrees, Figure 6B) and most of the strain applied to the material in the toe region can be used to recover the nanofibers shrinkage. In the case of bundles, the nanofibers alignment is lower (about 54% in the same range, Figure 6B) and a significant part of the applied strain has to be used to align the nanofibers and to recover the shrinkage. When both order and shrinkage of nanofibers are, respectively, imposed and recovered, the material can react to the applied strain with a higher stiffness. This point is the transition point. It is possible to notice that, in the case of aligned mats, when the TP was reached there was a huge increment in stiffness, measured by the elastic modulus E_TP_ (about 179.5 ± 5 MPa), and this fact can be explained by the recovery of the linear shape of a huge amount of nanofibers in the material. In the case of aligned bundles and HNES, at the TP the stiffness increment was less impressive but relevant (about 85.0 ± 3 MPa in the case of bundles, about 67.4 ± 4 MPa in the case of HNES) and the cause of this behavior can be related to the linear shape recovery of a limited numbers of nanofibers in the material.

The outlined behavior in the toe region and, in particular in the TP, has a relevant impact in mechanical behavior of the material in the final region. As highlighted in Figure 12, the greater is the E_TP_, the lesser is the strain of the knee point and the greater is the linear asymptotic modulus. In fact, the greater is the E_TP_, the greater is the number of nanofibers that recovered the linear shape and consequently, the sooner happens the transition at ε_Z,knee_ from the non-linear trend to the linear-asymptotic one.

Given the promising morphological and mechanical results obtained in the present work, the proposed HNES could be suitable for future applications in the fields of biomimetic materials, soft robotics, and tissue engineering. It has been reported that myocytes seeded onto 2D nanofibrous mats, showed a cellular growth and alignment along the fiber direction (Riboldi et al., 2005; Choi et al., 2008; Liao et al., 2008; Aviss et al., 2010; Mertens et al., 2014; Narayanan et al., 2016; Chernonosova et al., 2018; Yeo and Kim, 2018; Tang et al., 2019). In complex 3D scaffolds it is obviously more difficult to achieve cell infiltration into inner parts of the scaffold. However, recent results on electrospun hierarchical scaffolds, similar to HNES herein proposed, showed good cellular proliferation, infiltration, and elongation along the nanofibers direction in the whole structures, both in static and dynamic conditions (Sensini and Cristofolini, 2018; Apsite et al., 2019; Sensini et al., 2019b, 2020). Regarding potential soft robotics applications, several studies have investigated the combination of polyurethane coated with conjugated polymers such as polyaniline (Kang Gu et al., 2009), polypirrole Ebadi et al. (2019a, b) and PEDOT (Kwon et al., 2013), showing a good match between an electrostrictive and an elastomeric element. Polyurethane has been also used in blend with cellulose (Wang et al., 2018) or CNTs (Meng et al., 2019) in order to be actuated by moisture or electrical stimuli, respectively. Considering this literature background, in future works we will investigate the possibilities for our PU HNES to reproduce a new generation of bioinspired artificial muscles.

## Conclusion

In this work, we have successfully developed an innovative hierarchical electrospun muscle-inspired structure able to closely resemble the complex morphology of the skeletal muscular tissue. We demonstrated that by electrospinning it is possible to realize materials which mimic the alignment and geometry of nano- and micrometric arrangements like myofibrils, myofibers/fascicles and their surrounding membranes, up to the whole muscle. The mechanical characterization revealed slightly higher but comparable performances to the passive muscle ones, while maintaining a similar biomimetic non-linear behavior. Models have been introduced and successfully applied to study some mechanical behavior of the analyzed fibrous material. By means of the main characteristics of models it was possible to study the pre-toe region of aligned bundles and HNES. It was found that both bundles and HNES exhibited a very similar behavior which could be physically related to static frictional phenomena that influence the sliding between nanofibers. Moreover, it was possible to show the effect of the geometry of the nanofibers assembly on the mechanical behavior of materials with particular regard to the elastic properties. The biomimetic 3D structure developed in the present work is the basis for future developments in the field of muscle tissue engineering and of soft actuators, upon the introduction in the structure of active components, to achieve the realization of a highly biomimetic artificial muscle.

## Data Availability Statement

The datasets presented in this study can be found in online repositories. The names of the repository/repositories and accession number(s) can be found below: AMSActa Institutional Research Repository http://doi.org/10.6092/unibo/amsacta/6358.

## Author Contributions

CGo, AS, GF, CGu, MF, and AZ conceptualized the study. CGo, AS, GF, CGu, and AZ wrote the original draft and prepared the figures. CGo and AS produced the electrospun specimens and performed the morphological and mechanical characterization and analysis. CGo, AS, and GF optimized the electrospinning parameters. GF and CGu performed the thermal characterizations. AZ designed, developed, and analyzed the numerical modeling with the help of CGo and AS. MF, CGu, and AZ wrote, reviewed, and edited the draft. AZ and MF supervised and administrated the work and were responsible for the funding acquisition. All authors listed have made a substantial, direct and intellectual contribution to the work, and approved it for publication.

## Conflict of Interest

The authors declare that the research was conducted in the absence of any commercial or financial relationships that could be construed as a potential conflict of interest.
