# Supplementary material for: Biomimetic Hierarchically Arranged Nanofibrous Structures Resembling the Architecture and the Passive Mechanical Properties of Skeletal Muscles: A Step Forward Toward Artificial Muscle
Source: Front Bioeng Biotechnol. 2020 Jul 16;8:767. doi: 10.3389/fbioe.2020.00767 (PMC7379046; doi:10.3389/fbioe.2020.00767)
Supplement: Supplementary file 1 [file Table_1.DOCX]

## Table S1. The significance of differences between the net and apparent mechanical properties for the different samples (see tables in the draft) was assessed with a ratio paired parametric t-test.

|  | **σ_Y_**  **(MPa)** | **σ_F_**  **(MPa)** | **E**  **(MPa)** | **AS**  **(MPa)** | **L_Y_**  **(J/mm3)** | **L_F_**  **(J/mm3)** |
| --- | --- | --- | --- | --- | --- | --- |
| Random Mats | ****  (<0.0001) | ****  (<0.0001) | ****  (<0.0001) | ****  (<0.0001) | ****  (<0.0001) | ****  (<0.0001) |
| Random Bundles | ****  (<0.0001) | ****  (<0.0001) | ****  (<0.0001) | ****  (<0.0001) | ****  (<0.0001) | ****  (<0.0001) |
| Aligned Mats | ****  (<0.0001) | ****  (<0.0001) | ****  (<0.0001) | ****  (<0.0001) | ****  (<0.0001) | ****  (<0.0001) |
| Aligned Bundles | ****  (<0.0001) | ****  (<0.0001) | ****  (<0.0001) | ****  (<0.0001) | ****  (<0.0001) | ****  (<0.0001) |
| HNES | *  (0.0160) | *  (0.0160) | *  (0.0160) | *  (0.0160) | *  (0.0160) | *  (0.0160) |
