# Supplementary material for: Biomimetic Hierarchically Arranged Nanofibrous Structures Resembling the Architecture and the Passive Mechanical Properties of Skeletal Muscles: A Step Forward Toward Artificial Muscle
Source: Front Bioeng Biotechnol. 2020 Jul 16;8:767. doi: 10.3389/fbioe.2020.00767 (PMC7379046; doi:10.3389/fbioe.2020.00767)
Supplement: Supplementary file 2 [file Table_2.DOCX]

**Table S2.** The significance of differences between the apparent mechanical properties (and also the failure force and the yield and failure strain) for the samples with the same nanofibers orientation was assessed with an unpaired parametric t-test with Welch’s correction.

|  | **F**  **(N)** | **σ_Y_**  **(MPa)** | **σ_F_**  **(MPa)** | **ε_Y_**  **(%)** | **ε_F_**  **(%)** | **E**  **(MPa)** | **AS**  **(MPa)** | **L_Y_**  **(J/mm3)** | **L_F_**  **(J/mm3)** |
| --- | --- | --- | --- | --- | --- | --- | --- | --- | --- |
| Random  Mats vs Bundles | ****  (<0.0001) | ns  (0.1425) | ****  (<0.0001) | ns  (0.3283) | ****  (<0.0001) | ns  (0.0863) | ****  (<0.0001) | ns  (0.1005) | ****  (<0.0001) |
| Aligned  Mats vs Bundles | ****  (<0.0001) | ****  (<0.0001) | ****  (<0.0001) | ****  (<0.0001) | ****  (<0.0001) | ****  (<0.0001) | *  (0.0451) | ****  (<0.0001) | ****  (<0.0001) |
| Aligned  Bundles vs HNES | *  (0.028) | **  (0.0044) | *  (0.021) | ***  (0.0003) | **  (0.0039) | **  (0.0041) | *  (0.0498) | ***  (0.0007) | *  (0.0210) |
