# Supplementary material for: Biomimetic Hierarchically Arranged Nanofibrous Structures Resembling the Architecture and the Passive Mechanical Properties of Skeletal Muscles: A Step Forward Toward Artificial Muscle
Source: Front Bioeng Biotechnol. 2020 Jul 16;8:767. doi: 10.3389/fbioe.2020.00767 (PMC7379046; doi:10.3389/fbioe.2020.00767)
Supplement: Supplementary file 3 [file Table_3.DOCX]

**Table S3.** The significance of differences between the net mechanical properties for the samples with the same nanofibers orientation was assessed with an unpaired parametric t-test with Welch’s correction.

|  | σ_Y_  (MPa) | σ_F_  (MPa) | E  (MPa) | AS  (MPa) | L_Y_  (J/mm3) | L_F_  (J/mm3) |
| --- | --- | --- | --- | --- | --- | --- |
| Random  Mats vs Bundles | ns  (0.0878) | ****  (<0.0001) | ns  (0.2429) | ****  (<0.0001) | ns  (0.0980) | ****  (<0.0001) |
| Aligned  Mats vs Bundles | ****  (<0.0001) | ****  (<0.0001) | ****  (<0.0001) | **  (0.0033) | ****  (<0.0001) | ****  (<0.0001) |
| Aligned  Bundles vs HNES | ns  (0.1307) | *  (0.0124) | *  (0.0304) | *  (0.0199) | *  (0.0235) | *  (0.0124) |
